# Risk Factors for Early and Late Complications after Laparoscopic Sleeve Gastrectomy in One-Year Observation

**DOI:** 10.3390/jcm11020436

**Published:** 2022-01-15

**Authors:** Paulina Głuszyńska, Inna Diemieszczyk, Łukasz Szczerbiński, Adam Krętowski, Piotr Major, Hady Razak Hady

**Affiliations:** 1 1st Department of General and Endocrine Surgery, Medical University of Bialystok, 15-089 Bialystok, Poland; inna.demeschik@gmail.com (I.D.); hadyrazakh@wp.pl (H.R.H.); 2 Department of Endocrinology, Diabetology and Internal Medicine, Medical University of Bialystok, 15-089 Bialystok, Poland; lukasz.szczerbinski@umb.edu.pl (Ł.S.); adam.kretowski@umb.edu.pl (A.K.); 3 2nd Department of General Surgery, Jagiellonian University Medical College, 31-008 Krakow, Poland; majorpiotr@gmail.com

**Keywords:** bariatric surgery, complications, sleeve gastrectomy, risk factors

## Abstract

Background: Although laparoscopic sleeve gastrectomy (LSG) is considered a safe bariatric procedure in the treatment of obesity, it still involves a risk of developing postoperative complications. Knowledge of risk factors for possible complications would allow appropriate preoperative planning, optimization of postoperative care, as well as early diagnosis and treatment. The aim of this study was to evaluate risk factors for complications after laparoscopic sleeve gastrectomy. Methods: A retrospective study of 610 patients who underwent LSG at a tertiary institution were included in the study through retrospective analysis of the medical data. Complications were categorized as early (<30 days) and late (≥30 days) and evaluated according to the Clavien–Dindo Classification. Results: Early complications were observed in 35 patients (5.74%) and late complications occurred in 10 patients (1.64%). Independent risk factors of early complications after laparoscopic sleeve gastrectomy included hypercholesterolemia (OR 3.73; *p*-value = 0.023) and smoking (OR = 274.66, *p*-value < 0.001). Other factors that may influence the postoperative course are length of hospital stay and operation time. Smoking, peptic ulcer diseases and co-existence of hiatal hernia were found to be an independent predictors of late complications. Conclusions: Smoking is associated with the higher risk of both, early and late complications, while hypercholesterolemia with only <30 days complications after laparoscopic sleeve gastrectomy.

## 1. Introduction

Obesity is a public health concern that has reached the epidemic proportions as the prevalence of obesity has continued to increase worldwide, which has been leading to continuous rise in number of performed bariatric procedures [1,2]. Bariatric/metabolic surgery is considered to be the most effective method of morbid obesity treatment that provides satisfactory results not only in the terms of weight loss but also in the improvement or resolution of obesity-related diseases. Among all bariatric procedures, laparoscopic sleeve gastrectomy (LSG) is widely used worldwide in the surgical treatment of morbid obesity and it is considered to be minimally invasive and safe surgery with low complications and mortality rates. However, like every surgical intervention it carries the risk of the occurrence of complications and related mortality. The 30-day mortality rate after LSG is typically <0.2% [3,4]. The early complication rates after LSG are reported to be 5.4% to 7.3% and for severe complications 1.2% to 2.2% [5]. There are several risk factors considered predictive of overall complications after LSG that include older age, male sex, higher BMI, smoking and comorbidities such as: dyslipidemia, hypertension, diabetes, obstructive sleep apnea, liver disease, depression and others [6]. Identification of risk factors for complications after LSG would allow proper preoperative management of the patient and appropriate postoperative care. The aim of this study was to evaluate risk factors for early (in 30-day period) and late (≥30 days) complications after laparoscopic sleeve gastrectomy.

## 2. Materials and Methods

This is a retrospective cohort study of patients undergoing laparoscopic sleeve gastrectomy in the University Hospital at a tertiary institution between May 2012 and January 2020. Patients were qualified to surgical treatment of morbid obesity according to the Polish Guidelines on Metabolic and Bariatric Surgery [7]. Study was designed and conducted according with STROBE guidelines and the report of ISPOR task force [8,9]. Study inclusion criteria: patients, who underwent laparoscopic sleeve gastrectomy as a primary obesity surgery, no additional procedures during laparoscopic sleeve gastrectomy. Patients were excluded, when lack of necessary data occurred. Demographic and clinical data were gathered prospectively. The primary endpoint was to evaluate risk factors for early and late morbidity after LSG. All patients included in the study were divided into two groups: patients who experienced early and late complications and patients with no issues. Next, the comparison between two groups was performed taking into account several patient-related factors that could impact the occurrence of complications such as: gender, age, preoperative BMI, smoking within 2 months prior to the surgery, history of abdominal surgery, main comorbidities (arterial hypertension, type 2 diabetes mellitus, hypercholesterolemia, obstructive sleep apnea, chronic obstructive pulmonary disease, asthma, depression, non-alcoholic fatty liver disease). An early morbidity was defined as any deviation or adverse event apart from regular perioperative course, that required additional treatment and occurred within 30 days after laparoscopic sleeve gastrectomy. Late morbidity was defined as any deviation or adverse event apart from standard postoperative course, that required additional management and occurred ≥30 days and <12 months postoperatively. Complication findings that required postoperative management were captured according to the Clavien-Dindo classification [10].

### 2.1. Surgical Technique

All patients underwent laparoscopic sleeve gastrectomy performed by the same surgeon and two alternating assistants. The procedure started with the formation of pneumoperitoneum by the use of CO_2_ introduced to the peritoneal cavity by the Veress needle. The gas was insufflated until the intraabdominal pressure of 12–15 mmHg. The dissection of greater gastric curvature started approximately 6 cm proximally to the pylorus and was continued to the angle of His. The reduction in stomach volume was performed using a 36-Fr bougie and 60 mm linear staplers. At the end of the surgery, the leak test was performed with the use of air and 5% glucose solution.

### 2.2. Statistics

Data were analyzed using Statistica 13.5 software (StatSoft^®^, Tulsa, OK, USA). Continuous values were presented as means with standard deviations, or medians with interquartile ranges as appropriate. Chi-square Pearson’s test or chi-square test with Yates’ correction were used to compare between dichotomous data. Continuous variables were compared using T-student test or Mann-Whitney’s (for skewed variables) test. Univariate and multivariate logistic regression models were built in search of risk factors for morbidity after LSG. *p*-value ≤ 0.05 was considered to indicate statistical significance.

## 3. Results

The study group included 610 patients who had undergone LSG, 269 patients (44%) were men and 341 (56%) were women. Median age of patients was 43 (36–53) years and median BMI was 46.48 (42.24–51.53) kg/m^2^. The total number of patients scheduled for LSG at the analyzed time frame was 626, 16 patients were excluded from the study due to the lack of necessary data.

Overall early complications (<30 days) occurred in 35 patients (early morbidity rate 5.74%). All complications were categorized according to Clavien-Dindo classification and are presented in Table 1. Patients who developed more than one complication were classified to the highest grade of complications. Complications occurred in 15 men and 20 women (47%/53%) with median preoperative BMI = 45.36 kg/m^2^. Early complications were more likely to be developed by patients who had admitted to being active smokers (57.14% vs. 0.52%, *p*-value < 0.001). The most complications appeared in grade II and III. The most frequent type of complication in grade II was surgical infection and in grade III—gastric leak (six patients) and staple line bleeding (five patients). The analysis revealed two patients’ death due to the septic shock caused by gastric leak. The mortality rate in analyzed group was 0.33%. Comparison between patients with early morbidity and those without is presented in Table 2.

The risk factors associated with early complications were smoking, gastroesophageal reflux diseases, hypercholesterolemia, cholelithiasis, length of hospital stay and operation duration. As presented in Table 3, independent risk factors for developing early complications were hypercholesterolemia (OR 3.73; *p*-value = 0.023) and smoking (OR = 274.66, *p*-value < 0.001). 

The total incidence of late complications that occurred ≥30 days and <12 months after LSG was 1.64%. Late complications appeared in two men and eight women (20/80%). Five patients developed grade II complications according to Clavien-Dindo Classification (dumpling syndrome, optic neuropathy, staple-line ulcer and severe gastroesophageal reflux disease). Another five patients required surgical intervention due to hiatal hernia (one case) and trocar site hernia (four patients). All complications that occurred 30 days after the surgery are listed in Table 4. Comparison between patients with >30 days morbidity and those without is presented in Table 5. 

As shown in Table 6, independent risk factors for developing late complications (>30 days) include smoking (OR = 8.12, *p*-value = 0.008), peptic ulcer disease (OR = 6.75, *p*-value = 0.035) and co-existence of hiatal hernia (OR = 13.62, *p*-value = 0.043). 

## 4. Discussion

This study confirms that laparoscopic sleeve gastrectomy is a relatively safe bariatric procedure with 30-day morbidity rate being 5.74% and 30-day mortality rate of 0.3%. Our data reveals that tobacco smoking within 2 months before the bariatric surgery is associated with higher risk of both early and late complications, while hypercholesterolemia only increases the risk of early complications. Other factors that may influence postoperative course are co-existence of hiatal hernia, gastroesophageal reflux disease, cholelithiasis, peptic ulcer disease and operation-related factors such as operation duration and length of hospital stay.

Bariatric/metabolic surgery in morbidly obese patients is associated with the decrease in overall mortality in comparison with the control group treated conventionally [11]. However, obesity itself is known as a risk factor for developing surgical complications [12]. According to Sjöström et al., other factors that increase the risk of mortality during bariatric surgery are male gender, daily smoking and coexisting conditions such as diabetes, previous stroke and cancer, a history of myocardial infarction and lipid-lowering therapy [11]. Aminian et al. developed a risk calculator for perioperative complications after LSG that included male gender, BMI, presence of diabetes, history of congestive heart failure (CHF), steroid use for chronic condition, preoperative hematocrit level and preoperative serum total bilirubin level [13]. Besides the patient-related risk factors, there are also the procedure-related risk factors that may affect the incidence of complications after the surgical procedure such as procedure duration, occurrence of intraoperative complications, number of staple firings used and surgical experience of the surgeon [14,15].

In our study, we analyzed patient-related factors for developing early and late complications after laparoscopic sleeve gastrectomy. The research reveals that smoking is a factor significantly influencing the occurrence of <30 and >30 days after the surgery complications. Currently, no other individual factor has such a negative impact on human health as smoking [16]. According to Polish Guidelines on Metabolic and Bariatric Surgery smoking cessation is recommended at least 6 weeks before the surgical intervention [7,17]. Study conducted by Haskins et al. showed that tobacco use had a significant increase in prolonged intubation, reintubation, sepsis and length of hospital stay regardless of the type of the laparoscopic bariatric procedure. However, smoking did not lead to the increased risk of mortality for bariatric procedures [18]. Inadomi et al. conducted research to explore the relationship between smoking and short-term bariatric surgery outcomes. Their study revealed that risk-adjusted rate of severe complications among bariatric patients in the recent smoker group was significantly higher in comparison with the non-smoker group. However, the increased risk for developing severe complications applied only to the patients who had undergone Roux-en-Y gastric bypass (OR 1.34; 95% CI, 1.01–1.77) [19]. Another study conducted by Haskins et al. showed that smoking patients were more likely to experience a composite morbidity event (4.3% versus 3.7%, *p*-value = 0.04) and serious morbidity event (0.9% versus 0.6%, *p*-value = 0.003) [20]. The above analyses are in line with our study. However, Husain et al. did not identify any independent risk factor of severe complications after laparoscopic sleeve gastrectomy [6].

The precise mechanism by which smoking has a deleterious effect on surgical outcomes remains unknown. It is thought to be the result of both, long-term consequences of tobacco use and acute toxic results [21]. Smoking contributes to the damage of the gastric mucosa by increasing the apoptosis within the gastrointestinal tract. Increased apoptosis inhibits proliferation of mucosal cells that leads to impaired protective function and healing processes [22,23]. It has been also proven that nicotine, the major addictive agent in cigarettes, is a vasoconstrictor that reduces the blood flow, resulting in tissues ischemia. Additionally, it activates the sympathetic system and causes the release of catecholamines that leads to decrease in prostaglandins production and increases the platelets aggregation [24]. Moreover, carbon monoxide binds to hemoglobin reducing oxygen content that leads to tissue hypoxia [25]. The summarized impact of nicotine and carbon monoxide contributes to delay in all aspects of wound healing and leads to postoperative wound infection and gastric leak. Tobacco abuse also decreases the tension of lower esophageal sphincter what promotes retrograde flow of stomach contents to esophagus and causes symptoms of gastroesophageal reflux diseases (GERD) [26].

Abdominal obesity is a central point of metabolic syndrome (MS) components. It has been widely proven that laparoscopic sleeve gastrectomy has a considerable efficiency in the treatment of obesity-related diseases [27]. However, co-existing diseases may also cause an increased risk for the occurrence of complications. In our study, it has been demonstrated that hypercholesterolemia increases the risk of perioperative morbidity (OR 3.72; 95% CI, 1.20–11.56). Lorente et al. also proved that dyslipidemia is statistically relevant risk factor predicting overall and severe complications rate after bariatric surgery [28]. Additionally, hypercholesterolemia is a major cardiovascular risk factor that promotes the development of coronary artery disease [29]. Dorman et al. showed that patients with cardiac comorbidities that included: history of congestive heart failure, myocardial infarction or angina, previous coronary intervention or cardiac surgery are at higher risk of major postoperative events [30]. Diabetes is another commonly encountered diseases in patients who are candidates for bariatric surgery. A few studies have identified diabetes as a risk factor for severe complications: NSQIP study (OR = 2.04) and the Longitudinal Assessment of Bariatric Surgery study (LABS study) (OR = 1.46) [31,32]. Additionally, NSQIP study showed that postoperative complications were two times more likely to appear in patients after LSG (OR 2.06; 95% CI, 1.57–2.72) when compared to patients undergoing laparoscopic adjustable gastric banding (LAGB) [31]. In the recent study conducted by Guetta et al., patients with type 2 diabetes mellitus (T2DM) had significantly higher early complications rate than non-T2DM patients (13.3% vs. 7.0%, *p*-value = 0.01). Analysis of glycated hemoglobin level (HbA1c) as an independent variable showed that for every 1% elevation in HbA1c, there was an increase of 1.314 for early complications (*p*-value = 0.008; 95% CI, 1.07–1.61) and 1.407 for severe complications (*p*-value = 0.013; 95% CI, 1.07–1.84) [5]. These conclusions were not confirmed in our research.

Patients scheduled for bariatric surgery are considered to be at higher risk of perioperative risk due to the morbid obesity itself. However, there are also procedure-related factors that may increase the incidence of complications after laparoscopic sleeve gastrectomy. In our study, two factors: operation duration and length of hospital stay were found to have an impact on increased risk of perioperative issues, however they cannot be considered as independent factors for developing complications. In a study conducted by Sanni et al., the mean operative time for LSG was 93.3 ± 45.9 min (*p*-value < 0.0001). However, the operation time was not proved to have an influence on incidence of perioperative complications [31]. Additional procedures during the LSG, e.g., adhesiolysis or diaphragmatic crura repair may significantly extend the length of the procedure. However, an analysis performed by Major et al. did not show that additional procedures during laparoscopic sleeve gastrectomy are associated with the increased perioperative risk rate [14]. Husain et al. proved that long operation time (>120 min) for sleeve gastrectomy is a risk factor associated with severe complications after the procedure (III, IV and V grade according to Clavien-Dindo classification) [20]. Length of stay (LOS) after laparoscopic sleeve gastrectomy varies among different bariatric centers due to the non-identical discharge criteria. Fletcher et al. showed that patients with prolonged hospitalization defined as ≥3 days more often experienced organ space surgical site infection, pneumonia, pulmonary embolism, acute kidney injury, cardiac arrest and bleeding requiring transfusion. Their study also revealed statistically significant higher reoperation and readmission rates for hospitalizations ≥ 3 days when compared to 2 days (*p*-value < 0.001) [33]. Longer LOS was also a predictor of readmission in the study by Lois et al., in which they proved that patients with hospitalizations of more than 3 days were four times as likely to be readmitted than patients with one-day hospital stay after bariatric surgery (*p*-value < 0.001) [34].

The most common early complications that were observed in our study group were gastric leak and staple-line bleeding. Gastric leak is one the most serious and life-threatening complication that occurs in up to 5% of patients undergoing LSG [35]. The multicentre study conducted by Benedix et al. confirmed that male gender and BMI 50–50.9 kg/m^2^ are associated with significantly higher leak rates (2.5 vs. 1.6%, *p* = 0.02 and *p* < 0.01) [36]. Patients with gastric leak may be totally asymptomatic or present symptoms of septic shock, such as fever, abdominal pain, tachycardia, tachypnoea and peritonitis [37]. The management of gastric leak is dependent on the clinical status of the patient and it includes: conservative treatment (withholding food and fluids, intravenous hydration, broad spectrum antibiotheraphy, and proton pump inhibitor administration), endoscopic intervention such as implementation of endoprothesis or endoscopic double-pigtail catheter (EDPC) or surgical management (lavage and drainage of peritoneal cavity) [38,39].

The limitations of the present study are its non-randomized design, the relatively small sample of patients and short follow-up time, which was limited by the desire to provide complete data. However, this could result in underestimation of the real risk of developing >30 days complications after LSG. In addition, the real number of smoking patients may be greater as we suppose not everyone admitted to being an active smoker.

## 5. Conclusions

Laparoscopic sleeve gastrectomy is relatively safe procedure with low morbidity and mortality. However, it is important to recognize and optimize risk factors prior to the surgery in order to predict operative risk and improve surgical outcomes. The study confirms that tobacco smoking 2 months before the surgical procedure is associated with a higher risk of early and late complications. More attention should also be paid to co-existing diseases such as gastroesophageal reflux disease, peptic ulcer disease, hiatal hernia, hypercholesterolemia and cholelithiasis, that may influence the postoperative course.

## Figures and Tables

**Table 1:** Early (30-day) complications distribution according to Clavien-Dindo Classification.

Clavien-Dindo Grade and Type of Complication	Study Cohort (*n* = 610)
**Grade I**	1 (0.16%)
(Any deviation from the normal postoperative course without the need for pharmacological treatment or surgical, endoscopic and radiological interventions; allowed therapeutic regimens are: drugs as antiemetics, antipyretics, analgesics, diuretics and electrolytes and physiotherapy)	
Acute hypoxia	1
**Grade II**	13 (2.13%)
(Requiring pharmacological treatment with drugs other than such allowed for grade I complications; blood transfusions and total parenteral nutrition are also included)	
Staple-line bleeding with blood transfusion	1
Superficial thrombophlebitis	3
Superficial surgical site infection	2
Deep surgical site infection	4
Acute pancreatitis	1
Hepatitis	1
Severe gastroesophageal reflux disease	1
**Grade III**	13 (2.13%)
(Requiring surgical, endoscopic or radiological intervention)	
Gastric leak	6
Intraabdominal abscess	1
Gastric stenosis	1
Staple-line bleeding	4
Intraabdominal hematoma	1
**Grade IV**	6 (0.98%)
(Life-threatening complication (including central nervous system complications) requiring intermediate care/intensive care unit management)	

Pulmonary embolism	2
Portal and mesenteric vein thrombosis	1
Acute respiratory failure	2
Abdominal aorta dissection	1
**Grade V**	2 (0.33%)
(Death of a patient)	
Septic shock	2
**Total complications**	35 (5.74%)

**Table 2:** Comparison between patients with and without early (<30 days) morbidity.

Factor	<30 Days Morbidity	No Morbidity <30 Days	*p*-Value
Total, *n* (%)	35 (5.74%)	575 (94.26%)	n/a
Age, years, median (IQR)	46 (36–56)	43 (36–53)	0.286
Preoperative BMI, kg/m^2^, median (IQR)	45.36 (42.49–54.36)	46.57 (42.24–51.53)	0.410
Males/Females, *n* (%)	15/20 (43%/57%)	254/321 (44%/56%)	0.879
Smoking, *n* (%)	20 (57.14%)	3 (0.52%)	<0.001 *
Previous abdominal surgeries, *n* (%)	11 (31.43%)	146 (25.39%)	0.428
Gastroesophageal reflux disease, *n* (%)	5 (14.29%%)	33 (5.74%)	0.042 *
Chronic obstructive pulmonary disease, *n* (%)	1 (2.86%)	11 (1.91%)	0.696
Peptic ulcer disease, *n* (%)	2 (5.71%)	16 (2.78%)	0.631
Esophageal varices, *n* (%)	0	4 (0.70%)	n/a
Non-alcoholic fatty liver disease, *n* (%)	4 (11.43%)	43 (7.48%)	0.395
Hiatal hernia, *n* (%)	0	5 (0.87%)	n/a
Hypercholesterolemia, *n* (%)	8 (22.86%)	54 (9.39%)	0.023 *
Arterial hypertension, *n* (%)	15 (42.86%)	219 (38.09%)	0.573
Chronic heart ischemic disease, *n* (%)	1 (2.86%)	32 (5.57%)	0.762
Sleep apnea, *n* (%)	7 (20%)	83 (14.43%)	0.367
Depression, *n* (%)	4 (11.43%)	30 (5.22%)	0.120
Asthma, *n* (%)	1 (2.86%)	15 (2.61%)	0.929
Diabetes mellitus type 2, *n* (%)	8 (22.86%)	82 (14.26%)	0.164
Varicose veins, *n* (%)	7 (20%)	64 (11.13%)	0.112
Arthritis, *n* (%)	4 (11.43%)	42 (7.30%)	0.370
Cholelithiasis, *n* (%)	3 (8.57%)	8 (1.39%)	0.014 *
Kidney stones, *n* (%)	0	24 (4.17%)	n/a
Length of hospital stay, days, median (IQR)	3 (3–4)	3 (2–3)	<0.001 *
Operative time, min., median (IQR)	85 (60–110)	90 (60–110)	<0.001 *

Chi-square Pearson’s test or chi-square test with Yates’ correction were used to compare between dichotomous data. Continuous variables were compared using *t*-student test or Mann-Whitney’s (for skewed variables) test. * *p*-value < 0.05, significant difference.

**Table 3:** Factors influencing occurrence of early morbidity.

Factor	OR	95% CI	*p*-Value
Smoking	274.66	71.32–1057.70	<0.001 *
Hypercholesterolemia	3.72	1.20–11.56	0.023 *

Multivariate logistic regression model for risk factor of complications after LSG; * *p*-value < 0.05, significant difference.

**Table 4:** Late (≥30 days) complications distribution according to Clavien-Dindo Classification.

Clavien-Dindo Grade and Type of Complication	Study Cohort (*n* = 610)
**Grade I**	0
(Any deviation from the normal postoperative course without the need for pharmacological treatment or surgical, endoscopic and radiological interventions; allowed therapeutic regimens are: drugs as antiemetics, antipyretics, analgesics, diuretics and electrolytes and physiotherapy)	
**Grade II**	5 (0.82%)
(Requiring pharmacological treatment with drugs other than such allowed for grade I complications; blood transfusions and total parenteral nutrition are also included)	
Dumping syndrome	1
Severe gastroesophageal reflux	2
Staple-line ulcer	1
Optic neuropathy	1
**Grade III**	5 (0.82%)
(Requiring surgical, endoscopic or radiological intervention)	
Trocar site hernia	4
Hiatal hernia	1
**Grade IV**	0
(Life-threatening complication (including central nervous system complications) requiring intermediate care/intensive care unit management)	
**Grade V**	0
(Death of a patient)	
**Total complications**	10 (1.64%)

**Table 5:** Comparison between patients with and without late (≥30 days) morbidity.

Factor	≥30 Days Morbidity	No ≥30 Days Morbidity	*p*-Value
Total, *n* (%)	10 (1.64%)	600 (98.36%)	n/a
Age, years, median (IQR)	42 (34–44)	43 (36–53)	0.333
Preoperative BMI, kg/m^2^, median (IQR)	45.84 (39.63–51.38)	46.48 (42.24–51.53)	0.797
Males/Females, *n* (%)	2/8 (20%/80%)	267/333 (45%/56%)	0.122
Smoking, *n* (%)	3 (30%)	20 (3.33%)	<0.001 *
Previous abdominal surgeries, *n* (%)	4 (40%)	153 (25.52%)	0.499
Gastroesophageal reflux disease, *n* (%)	0	38 (6.33%)	n/a
Chronic obstructive pulmonary disease, *n* (%)	0	12 (2%)	n/a
Peptic ulcer disease, *n* (%)	2 (20%)	16 (2.67%)	0.023 *
Esophageal varices, *n* (%)	0	4 (0.67%)	n/a
Non-alcoholic fatty liver disease, *n* (%)	0	47 (7.83%)	n/a
Hiatal hernia, *n* (%)	1 (10%)	4 (0.67%)	0.139
Hypercholesterolemia, *n* (%)	0	62 (10.33%)	n/a
Arterial hypertension, *n* (%)	3 (30%)	231 (38.50%)	0.826
Chronic heart ischemic disease, *n* (%)	0	33 (5.50%)	n/a
Sleep apnea, *n* (%)	0	90 (15%)	n/a
Depression, *n* (%)	1 (10%)	33 (5.50%)	0.936
Asthma, *n* (%)	0	16 (2.67%)	n/a
Diabetes mellitus type 2, *n* (%)	2 (20%)	88 (14.67%)	0.982
Varicose veins, *n* (%)	0	71 (11.83%)	n/a
Arthritis, *n* (%)	0	46 (7.67%)	n/a
Cholelithiasis, *n* (%)	1 (10%)	10 (1.67%)	0.444
Kidney stones, *n* (%)	0	24 (4%)	n/a
Length of hospital stay, days, median (IQR)	3 (3–4)	3 (2–3)	0.779
Early morbidity, *n* (%)	2 (20%)	33 (5.50%)	0.204
Operative time, min., median (IQR)	80 (60–110)	85 (60–100)	0.078

Chi-square Pearson’s test or chi-square test with Yates’ correction were used to compare between dichotomous data. Continuous variables were compared using *t*-student test or Mann-Whitney’s (for skewed variables) test.; * *p*-value < 0.05, significant difference.

**Table 6:** Factors influencing occurrence of late morbidity.

Factor	OR	95% CI	*p*-Value
Smoking	8.12	1.72–38.35	0.008 *
Peptic ulcer disease	6.75	1.14–40.04	0.035 *
Hiatal hernia	13.62	1.08–171.21	0.043 *

Multivariate logistic regression model for risk factor of complications after LSG; * *p*-value < 0.05, significant difference.

## Data Availability

Not applicable.
